# Supplementary material for: Astrocytes release prostaglandin E2 to modify respiratory network activity
Source: eLife. 2017 Oct 6;6:e29566. doi: 10.7554/eLife.29566 (PMC5648524; doi:10.7554/eLife.29566)
Supplement: Supplementary file 1. [file elife-29566-supp1.docx]

**Supplementary Table 1. Number of experiments conducted**

| *Figure* | *Panel* | *Number of experiments* |
| --- | --- | --- |
| 1 | b | 5 |
|  | c | 7 |
|  | d | 6 |
|  | e | 6 |
|  | f | 6 |
|  | g | 5 |
|  | h | 6 |
|  | i | 6 |
|  | j | 5 |
|  | k | 5 |
|  | l | 5 |
|  | m | 6 |
| 2 | f | 19 |
|  | g | 22 |
