# Supplementary material for: Astrocytes release prostaglandin E2 to modify respiratory network activity
Source: eLife. 2017 Oct 6;6:e29566. doi: 10.7554/eLife.29566 (PMC5648524; doi:10.7554/eLife.29566)
Supplement: Supplementary file 2. [file elife-29566-supp2.docx]

**Supplementary Table 2. Statistical analyses and results**

| *Figure* | *Test used* | *Exact p-value* | *Degrees of freedom*  *&*  *F/t/z/R value* |
| --- | --- | --- | --- |
| 2b | Student’s t-test | 0.2841  0.5514 | DF = 40 |
| 2c and e | Student’s t-test | 0.0261 | DF = 40 |
| 2d | Student’s t-test | 0.1733  0.0941 | DF = 40 |
| 2h | Full factorial ANOVA | 0.821  0.688  0.716 | F=0.071  F=0.043  F=0.052  DFE=18 |
| 2j | Full factorial ANOVA | 0.416  0.237  0.0946 | F=0.064  F=0.065  F=0.038  DFE=21 |
| 3b | Student’s t-test | 0.0213  0.0278  0.0342  0.0413  0.0427 | DF=10, 10, 10, 7, 7 |
|  |  | 0.0147  0.0129  0.0385  0.0311  0.0406 | DF=11, 11, 11, 9, 9 |
| 3c | Full factorial ANOVA | 0.416  0.759 | F=0.029, DFE=6  F=0.063, DFE=7 |
| 4a | Student’s t-test | 0.0729  0.0271  0.0014  0.0298  0.194 | DF=7 |
| 4b | Student’s t-test | 0.017, 0.44  0.014, 0.71  0.29, 0.024 | DF=8 |
| 4c | Student’s t-test | 0.018, 0.41  0.74, 0.85  0.66, 0.70 | DF=7 |
